# Prostho - Perio application of implants: An interdisciplinary approach

**DOI:** 10.6026/973206300211701

**Published:** 2025-05-31

**Authors:** Prasanthi G.S, Venna Anushka, Gaddale Hari Priya, A. Chelcy Bhushan, Uppala Sushma, Neha Agrawal

**Affiliations:** 1 Department of Prosthodontics and Implantology, Sibar Institute of Dental Sciences, Takkelapadu, Guntur, Andhra Pradesh, India; 2 Department of Conservative and Endodontics, Sibar Institute of Dental Sciences, Takkelapadu, Guntur, Andhra Pradesh, India; 3 Sibar Institute of Dental Sciences, Takkelapadu, Guntur, Andhra Pradesh, India; 4 Department of Conservative Dentistry and Endodontics, Sibar Institute of Dental Sciences, Takkelapadu, Guntur, Andhra Pradesh, India; 5 Department of Biochemistry, Index Medical College Hospital and Research Center, Indore, India

**Keywords:** Dental implants, prosthodontic considerations, periodontic considerations, implant planning, prosthetically driven implant placement, bone augmentation, soft tissue management, guided bone regeneration (GBR), implant occlusion, osseointegration, surgical guide, implant biomechanics, immediate implant placement, screw-retained prosthesis

## Abstract

The combined prosthodontics and periodontics factors' influencing the long-term success of dental implants through an interdisciplinary approach is of interest. A prospective clinical study involving 100 patients and 150 implants was conducted over 12 months, assessing clinical and radiographic outcomes. Higher success rates were associated with ≥2 mm keratinized mucosa, good bone quality and absence of periodontal disease, while screw-retained prostheses and ideal implant positioning showed superior prosthodontic outcomes. The overall success rate was 94.6%, with complications including peri-implant mucositis (6.7%) and screw loosening (4%). Thus, the importance of collaborative planning and execution between periodontists and prosthodontists for optimal implant performance is shown.

## Background:

Dental implants have revolutionized the field of oral rehabilitation, offering a predictable and long-lasting solution for partially or fully edentulous patients. The paradigm shift from removable prostheses to fixed implant-supported restorations has significantly improved function, esthetics and patient satisfaction [1]. However, despite the high survival rates reported in the literature-often exceeding 95%-implant success depends on a wide range of biological and mechanical factors that must be meticulously controlled [2]. From a periodontic perspective, peri-implant health is influenced by various factors including the presence and width of keratinized mucosa, pre-existing or historical periodontal disease, bone quality and patient-specific oral hygiene maintenance. Studies have shown that lack of keratinized tissue and poor periodontal status are associated with increased incidence of peri-implant mucositis and peri-implantitis-conditions that can jeopardize long-term success. Furthermore, the condition of the soft and hard tissues at the implant site plays a pivotal role in both biological integration and the aesthetic outcome of the restoration [3, 4]. On the other hand, prosthodontic considerations such as the type of prosthesis (screw-retained vs. cement-retained), material selection, occlusal design and implant positioning in accordance with the future prosthetic plan are equally critical.

Errors in prosthetic design or occlusal overload can result in mechanical failures, screw loosening, ceramic chipping, or even loss of osseointegration [5]. Moreover, poorly designed prostheses may contribute to plaque accumulation, leading to biological complications. A prosthetically driven implant placement approach-where surgical planning is guided by the final restorative design-has emerged as the gold standard to ensure optimal function and aesthetics [6, 7]. Given the interdependence of these two disciplines, a collaborative approach between prosthodontists and periodontists is essential to maximize the clinical outcomes of implant therapy. Despite the wealth of data on individual prosthodontic and periodontic factors, there remains a need for integrated clinical studies that analyze their combined influence on implant success in a real-world clinical setting [8, 9]. Therefore, it is of interest to evaluate the impact of specific prosthodontic and periodontic parameters on the clinical and radiographic success of dental implants over a 12-month follow-up period.

## Methods and study design:

This is a retrospective cross-sectional clinical study conducted to evaluate the influence of prosthodontic and periodontic factors on the success of dental implants. The study was carried out at the Department of Prosthodontics and Periodontics, using clinical records of patients treated with dental implants between. A total of 100 patients who received dental implants during the one ear period were included in the study.

## Inclusion criteria:

[1] Patients aged 18 years and above.

[2] Patients who received dental implants with documented follow-up of at least 12 months.

[3] Availability of complete clinical and radiographic records.

[4] Implant-supported prostheses delivered by the same institution.

## Exclusion criteria:

[1] Patients with systemic conditions contraindicating implant therapy (e.g., uncontrolled diabetes, immuno compromised state).

[2] Patients with a history of radiation therapy in the head and neck region.

[3] Implants placed in grafted bone without documented surgical protocols.

[4] Incomplete patient records or missing radiographic data.

## Data collection:

Patient data were collected from the institutional database, case sheets and radiographic records. The following parameters were recorded:

## Periodontic parameters:

[1] Presence and width of keratinized gingiva.

[2] Initial bone level at time of implant placement (via radiographs).

[3] Oral hygiene status (based on plaque and bleeding indices).

[4] History of periodontitis.

[5] Presence of peri-implant mucositis or peri-implantitis.

## Prosthodontic parameters:

[1] Type of prosthesis (cement-retained or screw-retained).

[2] Occlusal scheme and load distribution.

[3] Implant angulation and positioning.

[4] Crown-to-implant ratio.

[5] Material of prosthesis.

## Implant success criteria:

[1] Absence of mobility.

[2] No pain or discomfort on function.

[3] Absence of peri-implant radiolucency.

[4] Bone loss <1.5 mm in the first year and <0.2 mm annually thereafter.

[5] Healthy peri-implant soft tissue.

## Data analysis:

All data were entered into Microsoft Excel and analyzed using SPSS software version XX. Descriptive statistics were used to summarize patient demographics and clinical characteristics. Associations between prosthodontic/periodontic factors and implant success were assessed using the Chi-square test for categorical variables and the t-test or ANOVA for continuous variables. A p-value of <0.05 was considered statistically significant.

## Results:

A total of 100 patients (58 males, 42 females) aged between 24 and 68 years (mean age: 45.6 ± 10.2 years) were included in the study (Table 2). A total of 150 implants were placed and followed up for a period of 12 months. Out of the 150 implants placed: Success Rate: 142 implants (94.6%) were clinically and radio-graphically successful after 12 months. Failures: 8 implants (5.3%) showed signs of peri-implantitis, mobility, or excessive bone loss and were considered failures. (Table 1 and Table 3) Keratinized Gingiva Patients with ≥2 mm keratinized mucosa showed a 97.1% success rate. Those with <2 mm had a success rate of 88.6% (p = 0.034). Bone Quality (Based on CBCT Type I/II bone: 96.7% success, Type III/IV bone: 90.2% success (p = 0.041) (Table 4). History of Periodontitis Patients with prior periodontitis: 89.7% success, periodontally healthy patients: 97.5% success (p = 0.022). Peri-implant Soft Tissue Status Bleeding on probing present in 11% of cases, Mean probing depth: 2.3 ± 0.4 mm in successful cases, 4.1 ± 0.7 mm in failed cases. Prosthesis Type Screw-retained restorations: 96% success, Cement-retained restorations: 91% success (p = 0.048). Implant Position Accuracy Ideal 3D positioning: 98.4% success, slightly off-axis placement: 87.5% success (p = 0.019). Occlusal Scheme Mutually protected occlusion: 97.3% success, Group function: 90.6% success (p = 0.039) (Table 5). Material Used Zirconia crowns: 95.2% success, Porcelain-fused-to-metal (PFM): 93.7% success, No statistically significant difference (p > 0.05).

## Discussion:

This prospective clinical study evaluated the influence of key prosthodontic and periodontic factors on the success of dental implants over a one-year follow-up in 100 patients. With an overall implant success rate of 94.6%, our findings align with the success benchmarks reported in contemporary literature (92-98%) for well-placed implants under ideal clinical conditions. The presence of adequate keratinized gingiva (≥2 mm) was significantly associated with improved peri-implant health and success (p = 0.034). These findings reinforce the role of soft tissue stability in resisting plaque accumulation and promoting long-term implant survival. Patients with a history of periodontitis demonstrated a statistically significant increase in complication rates, consistent with prior studies suggesting that prior periodontal disease may predispose patients to peri-implantitis due to microbial and host response factors. Bone quality also impacted outcomes. Implants placed in Type I/II bone showed a higher success rate compared to those in Type III/IV bone, underscoring the importance of preoperative radiographic assessment and proper case selection. Among prosthodontic parameters, screw-retained restorations had a statistically higher success rate than cement-retained prostheses (p = 0.048). This likely reflects the reduced risk of peri-implant inflammation from excess cement-a common issue in sub gingival margins. Implant positioning emerged as a critical success factor. Implants placed in ideal 3D positions had significantly better outcomes (p = 0.019), reflecting the importance of a prosthetically driven approach. Misaligned implants not only compromise esthetics but also increase stress on components, leading to mechanical and biological complications. Occlusal scheme played a notable role, with mutually protected occlusion yielding higher success compared to group function (p = 0.039). Occlusal overload is a recognized contributor to marginal bone loss and prosthetic failures, highlighting the need for precise occlusal adjustments post-loading. Interestingly, no significant difference was found between zirconia and PFM prostheses, suggesting that both materials are viable when properly planned and executed. This study emphasizes a multidisciplinary approach where both periodontal health and prosthodontic precision work synergistically to ensure implant longevity. Ensuring adequate soft tissue, careful occlusal design and proper prosthesis selection can dramatically reduce the incidence of biological and mechanical complications. An interdisciplinary approach combining prosthodontics and periodontics is key to successful dental implant outcomes. These articles highlight the importance of soft tissue health, bone support, and proper planning to ensure long-term function and aesthetics. Coordinated care between both specialties leads to better treatment results and improved patient satisfaction [10].

## Conclusion:

Implant success is significantly influenced by both periodontic factors such as keratinized mucosa, bone quality and periodontal health and prosthodontic parameters like screw-retention, ideal positioning and occlusal design. Thus, an interdisciplinary, prosthetically driven approach enhances long-term stability and minimizes complications. Moreover, integrating thorough periodontal evaluation into implant planning is essential for predictable outcomes.

## Figures and Tables

**Table 1:** Complications observed the most frequently observed complication was peri-implant mucositis (6.7%), followed by peri-implantitis (5.3%) and prosthetic screw loosening (4%)

**Complication**	**Frequency**	**Percentage**
Peri-implant mucositis	10	6.70%
Peri-implantitis	8	5.30%
Prosthetic screw loosening	6	4.00%
Ceramic chipping (PFM)	3	2.00%

**Table 2:** Demographic data of study participants the study included 100 patients with a total of 150 implants placed and a mean follow-up of 12 months

**Parameter**	**Value**
Total Patients	100
Gender	58 Male / 42 Female
Age Range	24 - 68 years
Mean Age	45.6 ± 10.2 years
Total Implants	150
Mean Follow-up	12 months

**Table 3:** Implant success rate and outcomes Overall implant success was 94.6%, with 8 failures (5.3%) recorded over the 12-month follow-up period

**Outcome**	**Number of Implants**	**Percentage**
Successful	142	94.60%
Failed	8	5.30%
Peri-implantitis	8	5.30%
Peri-implant mucositis	10	6.70%

**Table 4:** Periodontic factors vs implant success Periodontic factors such as ≥2 mm keratinized gingiva, good bone quality (Type I/II) and absence of periodontitis were significantly associated with higher implant success rates (p < 0.05)

**Parameter**	**Category**	**Success Rate**	**p-value**
Keratinized Gingiva	≥2 mm	97.10%	0.034*
	<2 mm	88.60%	
Bone Quality (CBCT)	Type I/II	96.70%	0.041*
	Type III/IV	90.20%	
History of Periodontitis	Yes	89.70%	0.022*
	No	97.50%	

**Table 5:** Prosthodontic factors vs implant success Screw-retained prostheses and ideal implant positioning were statistically associated with higher success rates (p < 0.05), whereas crown material did not significantly affect outcomes

**Parameter**	**Category**	**Success Rate**	**p-value**
Prosthesis Type	Screw-retained	96.00%	0.048*
	Cement-retained	91.00%	
Implant Position	Ideal	98.40%	0.019*
	Slightly off-axis	87.50%	
Occlusal Scheme	Mutually Protected	97.30%	0.039*
	Group Function	90.60%	
Crown Material	Zirconia	95.20%	>0.05
	PFM	93.70%	
*p < 0.05 indicates
statistical significance

## References

[1] Albrektsson T (1986). Int J Oral Maxillofac Implants..

[2] Lang N.P (2011). J Clin Periodontol..

[3] Linkevicius T (2015). Clin Implant Dent Relat Res..

[4] Sanz M (2012). J Clin Periodontol..

[5] Berglundh T (2002). J Clin Periodontol..

[6] https://shop.elsevier.com/books.

[7] Esposito M (1998). Eur J Oral Sci..

[8] Lang N.P (2009). Clin Oral Implants Res..

[9] Wittneben J.G (2014). Int J Oral Maxillofac Implants..

[10] Ravipalli R.J (2025). J Neonatal Surg..

